# Nivolumab Versus Sorafenib as First-Line Therapy for Advanced Hepatocellular Carcinoma: A Cost-Effectiveness Analysis

**DOI:** 10.3389/fphar.2022.906956

**Published:** 2022-07-19

**Authors:** Yan Li, Xueyan Liang, Huijuan Li, Tong Yang, Sitong Guo, Xiaoyu Chen

**Affiliations:** Department of Pharmacy, Guangxi Academy of Medical Sciences and the People’s Hospital of Guangxi Zhuang Autonomous Region, Nanning, China

**Keywords:** nivolumab, sorafenib, cost-effectiveness, advanced hepatocellular carcinoma, partitioned survival model

## Abstract

**Objective:** Nivolumab improves overall survival (OS) and is associated with fewer adverse events than sorafenib for the treatment of advanced hepatocellular carcinoma (aHCC). However, the cost-effectiveness of nivolumab compared with sorafenib treatment for aHCC remains unclear. This study evaluated the cost-effectiveness of nivolumab and sorafenib in the treatment of aHCC.

**Materials and methods:** A partitioned survival model that included three mutually exclusive health states was used to evaluate the cost-effectiveness of nivolumab and sorafenib for treating aHCC. The clinical characteristics and outcomes of the patients in the model were obtained from the CheckMate 459. We performed deterministic one-way sensitivity and probabilistic sensitivity analyses to evaluate the robustness of the model. Subgroup analyses were also performed. Costs, life-years, quality-adjusted life-years (QALYs), incremental cost-effectiveness ratio (ICER), incremental net health benefits (INHB), and incremental net monetary benefits (INMB) were measured.

**Results:** The base case analysis showed that compared with sorafenib, treatment with nivolumab was associated with an increment of 0.50 (2.45 vs. 1.95) life-years and an increment of 0.32 (1.59 vs. 1.27) QALYs, as well as a $69,762 increase in cost per patient. The ICER was $220,864/QALY. The INHB and INMB were −0.15 QALYs and −$22,362 at a willingness-to-pay (WTP) threshold of $150,000/QALY, respectively. The probabilistic sensitivity analysis demonstrated that the probability of nivolumab being cost-effective was only 10.38% at a WTP threshold of $150,000/QALY. The model was most sensitive to the costs of sorafenib and nivolumab according to the one-way sensitivity analysis. When the price of sorafenib exceeded $0.93/mg or nivolumab was less than $24.23/mg, nivolumab was more cost-effective. The subgroup analysis illustrated that the probability of cost-effectiveness was >50% in the Barcelona Clinic Liver Cancer Stage B subgroups for nivolumab at a WTP threshold of $150,000/QALY. This study also showed that the probability of cost-effectiveness was <50% in most subgroups.

**Conclusion:** Nivolumab was not cost-effective, although it was associated with better clinical benefit and a favorable safety profile for the treatment of aHCC compared with sorafenib from the third-party payer perspective in the United States. If the price of nivolumab is substantially reduced, favorable cost-effectiveness can be achieved among patients with aHCC.

## Introduction

Hepatocellular carcinoma (HCC) comprises 75–85% of primary liver cancer cases, and is the fourth-leading cause of annual cancer deaths worldwide (Gordan et al., 2020). Although diagnosis of HCC at early stages will possibly obtain curative treatments, such as resection or liver transplantation, only 30–40% of patients with HCC receive an early diagnosis (Forner et al., 2018). Most patients with HCC are diagnosed at an advanced stage and have a poor prognosis (Park et al., 2015). Therapies for advanced HCC (aHCC) include sorafenib (multikinase inhibitors) that increase median overall survival (OS) to 12.3 months (Kudo et al., 2018). However, sorafenib is associated with a high proportion of drug-related adverse events (AEs), and outcomes remain poor. Consequently, treatment options for aHCC remain very limited, and the prognosis is poor.

For the past few years, immunotherapy for many tumor types, including HCC, has received great attention (Zakeri et al., 2022). Nivolumab, an anti-programmed cell death protein-1 (PD-1) antibody, inhibits immune checkpoint signaling (Cheung et al., 2021). Nivolumab treatment for several tumor types, such as melanoma (Weber et al., 2015) and non-small cell lung cancer (Borghaei et al., 2015; Brahmer et al., 2015), improves survival compared with chemotherapy. The CheckMate-040 trial demonstrated the efficacy and safety of nivolumab as second-line therapy for aHCC (El-Khoueiry et al., 2017). With the increasing economic burden of healthcare costs, value-based oncology is drawing more attention; therefore, nivolumab has garnered great attention as a leading immunotherapy approach (Pei et al., 2021). Nivolumab has been approved in many countries for the treatment of sorafenib-receiving patients with aHCC, relying on the results of the CheckMate-040 trial (El-Khoueiry et al., 2017).

Recently, a CheckMate 459 phase 3 randomized multicenter clinical trial (Yau et al., 2022) reported the clinical activity and favorable safety of nivolumab as a first-line treatment for aHCC compared with sorafenib. The results revealed that the median follow-up for OS was 15.2 and 13.4 months for nivolumab and sorafenib treatment, respectively. In addition, the median OS was 16.4 and 14.7 months for nivolumab and sorafenib treatment, respectively. Although these increases were not statistically significant, they suggested that nivolumab might offer a potentially better survival chance. Moreover, the most common adverse event (AE) was palmar-plantar erythrodysesthesia, the incidence of which was lower following nivolumab treatment (<1%) than sorafenib treatment (14%). Thus, nivolumab may be a potential first-line alternative treatment for aHCC. However, with this convincing clinical outcome, the concomitant high drug price has been in the spotlight. To the best of our knowledge, no cost-effectiveness analyses comparing nivolumab with sorafenib for aHCC have been published. Cost-effectiveness analyses are helpful for optimally distributing limited healthcare resources to clinicians and decision-makers; it is necessary to perform a cost-effectiveness analysis to compare the efficacy and cost of nivolumab. Thus, from the third-party payer perspective in the United States (USA), this study evaluated the cost-effectiveness of nivolumab as a first-line therapy for aHCC.

## Materials and Methods

### Patients and Intervention

This study was performed in accordance with the Consolidated Health Economic Evaluation Reporting Standards (CHEERS, Supplementary Table S1) (Husereau et al., 2022). According to the People’s Hospital of Guangxi Zhuang Autonomous Region, since publicly available data from the literature and open database were used to conduct this study rather than individual patient-level data, institutional review board review and informed consent were not required nor obtained.

Hypothetical target patients with aHCC were obtained from the CheckMate 459 randomized clinical trial (Yau et al., 2022). Included patients were adults (aged ≥18 years), with a performance status of 0 or 1 on the Eastern Cooperative Group scale; no previous systemic therapy; no previous radiotherapy within 4 weeks before study drug commencement; and had to have adequate hematological, hepatic, renal, and cardiac function. According to the CheckMate 459 trial report (Yau et al., 2022), patients assigned to the nivolumab group received 240 mg nivolumab intravenously every 2 weeks, and those in the sorafenib group received 400 mg of sorafenib orally twice daily. When the disease progressed or unacceptable AEs occurred, alternate therapies were administered.

### Model Structure

**FIGURE 1:**
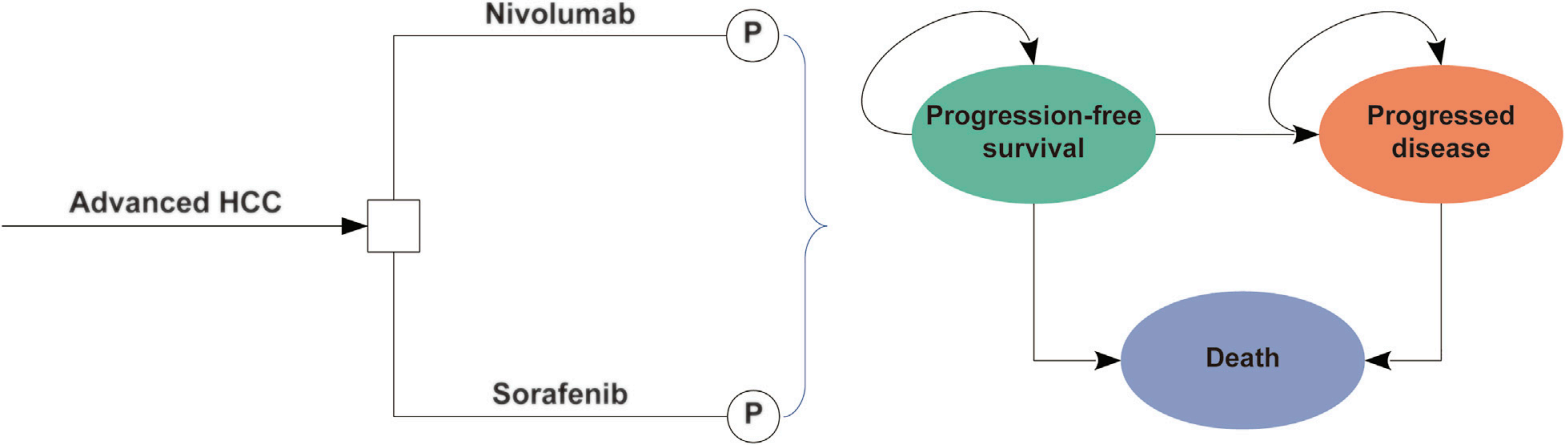
The partitioned survival model consisting of three discrete health states. Abbreviations: HCC, hepatocellular carcinoma; P, partitioned survival model.

In this study, we performed an economic evaluation and constructed a partitioned survival model based on three mutually exclusive health states: progression-free survival (PFS), progressive disease (PD), and death (Figure 1) (Williams et al., 2017). The time horizon was 10 years, and more than 98% patients died in both treatment arms. The cycle length was 1 week. In the model, the proportions of patients with OS and PFS were determined based on the results of the CheckMate 459 trial (Yau et al., 2022). The area under the OS curve was evaluated for the proportion of patients alive, the area under the PFS curve was evaluated for the proportion of patients alive with PFS, and the difference between the OS and PFS curves was evaluated for the proportion of patients alive with PD.

### Clinical Data Inputs

The patients with aHCC in the nivolumab and sorafenib groups were determined based on the results of the CheckMate 459 trial (Yau et al., 2022). Both OS and PFS were extrapolated beyond the trial’s follow-up time horizon that was calculated based on the algorithm created by Guyot et al. (2012). The Kaplan–Meier (K-M) survival curves of OS and PFS data were obtained from the trial using GetData Graph Digitizer version 2.26 (Get Data Graph Digitizer, 2022) to extract the individual patient data points. These data points were then used to fit the following parametric survival functions: exponential, Weibull, gamma, log-normal, Gompertz, log-logistic, and generalized gamma distributions. Subsequently, according to the value of Akaike information criterion (AIC) and Bayesian information criterion (BIC), the best-fit parametric models for the reconstructed K-M survival curves were selected. The results of the survival functions and parametric models of nivolumab and sorafenib treatment are shown in Table 1, and the goodness-of-fit results are shown in Supplementary Table S2. Log-normal was used to fit the OS and PFS K-M curves of nivolumab and sorafenib, respectively (Supplementary Figure S1). The key clinical input data are listed in Table 1.

**TABLE 1:** Key model inputs.

Parameter	Expected value (range)	Distribution	Source
Clinical input			
Survival model for sorafenib			
Log-normal model for PFS^a^	Log-mean = 2.98, log-SD = 0.88	ND	Yau et al. (2022)
Log-normal model for OS^a^	Log-mean = 4.07, log-SD = 1.13	ND	Yau et al. (2022)
Survival model for nivolumab^b^			
Log-normal model for PFS^a^	Log-mean = 3.05, log-SD = 1.10	ND	Yau et al. (2022)
Log-normal model for OS^a^	Log-mean = 4.23, log-SD = 1.30	ND	Yau et al. (2022)
HR for PFS associated with nivolumab vs. sorafenib	0.93 (0.79–1.10)	Log-normal: log-mean = −0.073, log-SD = 0.084	Yau et al. (2022)
HR for OS associated with nivolumab vs. sorafenib	0.85 (0.72–1.02)	Log-normal: log-mean = −0.16, log-SD = 0.089	Yau et al. (2022)
Utility input			
Utility of PFS	0.76 (0.57–0.95)	Beta: *α* = 4.7, *β* = 1.5	(Shlomai et al. (2018))
Utility of PD	0.68 (0.54–0.82)	Beta: *α* = 29, *β* = 13.6	(Shlomai et al. (2018))
Disutility due to AEs			
Grade 1 and 2	0.01 (0.008–0.012)	Beta: *α* = 18, *β* = 1283.2	(Amdahl et al. (2016))
Grade 3 and higher	0.16 (0.12–0.20)	Beta: *α* = 36, *β* = 193	(Amdahl et al. (2016))
Cost input			
Nivolumab per 200 mg^b^	5,849 (4,387–7,311)	Gamma: *α* = 53.41, *β* = 109.5	(Centers for Medicare & Medicaid Services, (2022); RED BOOK online, (2022))
Sorafenib per 200 mg^b^	158 (127–212)	Gamma: *α* = 39.09, *β* = 131.24	RED BOOK online, (2022)
Second-line treatment in nivolumab arm	5,131 (1,311–6,739)	Gamma: *α* = 53, *β* = 68.97	(Yau et al. (2022); Centers for Medicare & Medicaid Services, (2022); RED BOOK online, (2022))
Second-line treatment in sorafenib arm	3,656 (2,045–4,640)	Gamma: *α* = 99.88, *β* = 1.58	(Yau et al. (2022); Centers for Medicare & Medicaid Services, (2022); RED BOOK online, (2022))
Subsequent best supportive care per patient^c^	39,875 (29,906–49,843)	Gamma: *α* = 16, *β* = 2492.19	Soto-Perez-de-Celis et al. (2019)
Follow-up and monitoring per cycle			
Patients with PFS^d^	212 (159–265)	Gamma: *α* = 16, *β* = 13.25	(Su et al. (2021))
Patients with PD^d^	246 (185–308)	Gamma: *α* = 16, *β* = 15.38	(Su et al. (2021))
Drug administration per unit	80 (60–100)	Gamma: *α* = 16, *β* = 5	(Amdahl et al. (2016))
Terminal care per patient^d^	8,488 (6,366 to 10,610)	Gamma: *α* = 16, *β* = 530.5	(Soto-Perez-de-Celis et al. (2019))
Costs of AEs (more than grade 3)			
Nivolumab	503.94 (374.37–635.86)	Gamma: *α* = 53, *β* = 9.43)	(Patel et al. (2011); Barzey et al. (2013); Kacker et al. (2013); Wilson et al. (2017))
Sorafenib	3042.80 (2269.87–3822.95)	Gamma: *α* = 53, *β* = 56.97)	(Patel et al. (2011); Barzey et al. (2013); Kacker et al. (2013); Wilson et al. (2017))

Abbreviations: PFS, progression-free ; OS, overall survival; HR, hazard ratio; ND, not determined; PD, progressed disease; AEs, adverse events.

a Only expected values are presented for these survival model parameters.

b Treatment with nivolumab and sorafenib continued until disease progression or unacceptable toxicity.

c Overall total cost per patient regardless of treatment duration.

d These costs were assumed to be continued until the health state transitioned.

### Cost

Direct medical costs were evaluated, including the cost of acquiring drugs, attributed to the cost of the patient’s health state, cost of supportive care, cost of terminal care, and AE-related costs (Table 1). The prices of acquiring drugs were collected from public databases (Centers for Medicare & Medicaid Services, 2022; RED BOOK online, 2022; Yau et al., 2022). The monitoring costs for patients with PFS and PD were $212 and $246 per cycle, respectively (Su et al., 2021). After the disease progression, about 57% of patients in the nivolumab group and 71% patients in the sorafenib group received second-line treatment according to published reports (Yau et al., 2022). The costs related to subsequent supportive care and terminal care were $39,875 and $8,488 per patient, respectively (Soto-Perez-de-Celis et al., 2019). The costs associated with severe adverse event (SAE, grade ≥3) management were sourced from the literature (Supplementary Table S3) (Patel et al., 2011; Barzey et al., 2013; Kacker et al., 2013; Hornberger et al., 2015; Wilson et al., 2017). All costs were adjusted to 2021 US dollars and were inflated to 2021 monetary values based on the Medical-Care Inflation data obtained from Tom’s Inflation Calculator (Tom’s Inflation Calculator, 2022), and these values are shown in Table 1.

### Effectiveness

Health utility scores were assigned on a scale from 0 (death) to 1 (perfect health). Considering that health utilities for PFS and PD were not provided in CheckMate 459, we used health utility scores from the published literature (Shlomai et al., 2018). The utilities of PFS and PD related to aHCC were 0.76 and 0.68, respectively, which were obtained from an analysis of cost-effectiveness evaluating patients with HCC (Shlomai et al., 2018). The disutility values associated with AEs were also obtained from the literature (Amdahl et al., 2016).

### Base Case Analysis

The incremental cost-effectiveness ratio (ICER), presented as the incremental cost per additional quality-adjusted life-years (QALYs) gained, was examined. Based on the published literature (Su et al., 2021), the WTP threshold in the United States was $150,000. When the ICER was lower than the WTP threshold ($150,000/QALY), cost-effectiveness was assumed according to the recommendations (Neumann et al., 2014). A 3% annual discount rate was derived for costs and utility outcomes (Sanders et al., 2016). We also calculated the incremental net health benefits (INHB) and incremental net monetary benefits (INMB) (Su et al., 2021). The INHB and INMB are computed according to the following formulas: INHB(λ) = (*μ*_*E*1_ − *μ*_*E*0_) − (*μ*_*C*1_ − *μ*_*C*0_)/λ = Δ*E* − Δ*C*/λ and INMB(λ) = (*μ*_*E*1_ − *μ*_*E*0_) × λ − (*μ*_*C*1_ − *μ*_*C*0_) = Δ*E* × λ − Δ*C*, where *μ*_*Ci*_ and *μ*_*Ei*_ were the cost and utility of nivolumab (*i* = 1) or sorafenib (*i* = 0), respectively, and λ was the WTP threshold.

### Sensitivity Analyses

In this study, we performed one-way sensitivity analysis to identify significantly sensitive variables and evaluated the robustness of the results. One-way sensitivity analyses were performed based on different variables, such as costs and utilities, and the uncertainty of each variable was calculated according to 95% confidence intervals (CIs) reported in the literature or estimated by assuming a 25% variation from the fundamental parameters (Table 1). We also conducted probabilistic sensitivity analysis with 10,000 iterations, for which Monte Carlo simulations were used. All parameters determined a suitable distribution (Vaidya et al., 2014). A gamma, log-normal, and beta distributions were assigned to the cost parameters, hazard ratios (HRs), and proportion, probability, and preference value parameters, respectively. Subsequently, a cost-effectiveness acceptability curve was constructed to illustrate the possibility that nivolumab or sorafenib would be valuable at various WTP levels/QALYs gain.

### Subgroup Analyses

Subgroup analyses were performed to explore the uncertainty of the outcomes caused by different patient characteristics. Subgroup analyses were constructed for the different subgroups derived from CheckMate 459 by varying the HR for OS, including geographical region, age, Barcelona clinic liver cancer stage, Child–Pugh score, disease cause, vascular invasion or extrahepatic spread, baseline alpha-fetoprotein, and baseline tumor-cell PD-L1 expression (Yau et al., 2022). Statistical analyses in this study were performed with hesim and heemod packages in R, version 4.0.5, 2021 (R Foundation for Statistical Computing).

## Results

### Base Case Analysis

For base case analysis of the total patients with aHCC, nivolumab led to an increased effectiveness of 0.32 QALYs and 0.50 overall life-years, with an additional cost of $69,762 compared with the sorafenib arm. The corresponding ICER was $220,864/QALY. Furthermore, the INHB and INMB of nivolumab were −0.15 QALYs and −$22,362, respectively, at a $150,000/QALY WTP threshold compared with sorafenib (Table 2).

**TABLE 2:** Summary of cost and outcome results in the base-case analysis.

Factor	Nivolumab	Sorafenib	Incremental change
Cost, $			
Drug^a^	366,661	299,477	67,184
Nondrug^b^	23,637	21,059	2,578
Overall	390,298	320,536	69,762
Life-years			
Progression-free	0.74	0.56	0.18
Overall	2.45	1.95	0.50
QALYs	1.59	1.27	0.32
ICER, $			
Per life-year	NA	NA	138,514
Per QALY	NA	NA	220,864
INHB, QALY, at threshold 150,000^a^	NA	NA	−0.15
INMB, $, at threshold 150,000^a^	NA	NA	−22,362

Abbreviations: ICER, incremental cost-effectiveness ratio; INHB, incremental net health benefit; INMB, incremental net monetary benefit; NA, not applicable; QALYs, quality-adjusted life-years.

a Compared with sorafenib.

b Nondrug cost includes the costs of adverse event management, subsequent best supportive care per patient, and follow-up care covering physician monitors, drug administration, and terminal care.

### Sensitivity Analysis

The results of the one-way sensitivity analyses illustrated that the primary drivers of the model outcome included the cost of sorafenib and nivolumab, as well as their utility for PD and PFS. This is because these factors have a considerable impact on the ICER (Supplementary Figure S2). The remaining parameters, such as HR for PFS and OS, were only moderately or weakly related to the outcomes and were not related to ICER exceeding the threshold of $150,000/QALY. We also evaluated the relevance of these key variables with the ICER between nivolumab and sorafenib. When the price of sorafenib exceeded $0.93/mg or nivolumab was less than $24.23/mg, nivolumab was cost-effective at a WTP threshold of $150,000/QALY (Supplementary Figure S3).

**FIGURE 2:**
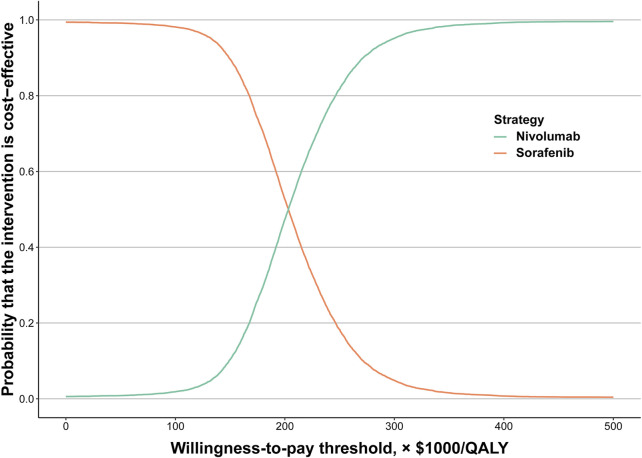
Acceptability curves of cost-effectiveness for nivolumab versus sorafenib. Abbreviations: QALY, quality-adjusted life-year

The results of the probabilistic sensitivity analysis were displayed by the cost-effectiveness acceptability curve (Figure 2). The probability of nivolumab being cost-effective increased as the WTP thresholds increased. Compared to sorafenib (89.62%), the probability of nivolumab being considered cost-effective was only 10.38% at a WTP threshold of $150,000/QALY for the total population. However, at a WTP threshold of $300,000/QALY, the probability of nivolumab and sorafenib being considered cost-effective was 95.14 and 4.86%, respectively.

### Subgroup Analysis

The subgroup analysis was performed by varying the HRs for OS. Compared with sorafenib, nivolumab was associated with higher HRs in the subgroups of Barcelona clinic liver cancer stage B and without vascular invasion or extrahepatic spread [hazard ratio: 1.35 (95% CI: 0.86–2.11) and 1.14 (95% CI: 0.81–1.62), respectively]; hence, the results of subgroup analysis illustrated that nivolumab had >50% probability of being considered cost-effective in the Barcelona clinic liver cancer stage B subgroup at a WTP threshold of $150,000/QALY (Table 3). The probability of nivolumab being considered cost-effective was <50% in most of the subgroups.

**TABLE 3:** Summary of subgroup analyses obtained by varying the hazard ratios (HRs) for overall survival.

Subgroup	Unstratified HR for OS (95% CI)	Change in cost, $^a^	Change in QALYs^a^	ICER, $/QALY	Cost-effectiveness probability of nivolumab, %, at threshold 150,000
Geographical region					
Asia	0.74 (0.56–0.98)	69,762	0.316	220,864	0.044
Non-Asia	0.92 (0.74–1.14)	22,805	0.123	185,040	0.44
Age, years					
<65	0.80 (0.63–1.02)	−71,081	−0.327	217,172	0.44
≥65	0.88 (0.68–1.12)	37,651	0.186	202,428	0.46
Barcelona Clinic Liver Cancer stage					
A	0.49 (0.17–1.40)	268,861	1.163	231,118	1.165
B	1.35 (0.86–2.11)	−83,919	−0.328	255,952	82.66
C	0.78 (0.65–0.95)	80,309	0.366	219,238	0.74
Child-Pugh score					
5	0.89 (0.72–1.10)	33,831	0.170	199,182	0.56
6	0.79 (0.57–1.09)	75,648	0.347	218,253	0.61
Disease cause					
Hepatitis C virus infected	0.71 (0.49–1.01)	115,754	0.516	224,272	0.87
Hepatitis B virus infected	0.77 (0.56–1.05)	85,065	0.386	220,140	0.51
Uninfected	0.95 (0.74–1.22)	12,396	0.079	156,425	0.61
Vascular invasion or extrahepatic spread					
Yes	0.74 (0.61–0.90)	-99,934	−0.449	222,440	0.65
No	1.14 (0.81–1.62)	41,657	0.149	279,143	49.76
Baseline alpha-fetoprotein, μg/L					
<400	0.98 (0.78–1.24)	2,562	0.038	67,990	0.5
≥400	0.67 (0.51–0.88)	138,456	0.612	226,201	1.1
Baseline tumor-cell PD-L1 expression					
≥1%	0.80 (0.54–1.19)	71,081	0.327	217,172	0.53
<1%	0.84 (0.69–1.02)	53,703	0.254	211,556	0.42

Abbreviations: HR, hazard ratio; ICER, incremental cost-effectiveness ratio; OS, overall survival; PD-L1, programmed cell death ligand 1; QALY, quality-adjusted life-year; WTP, willingness-to-pay.

a HR for OS represents the HR of nivolumab vs. sorafenib for OS; change in cost and change in QALYs represent the results of nivolumab minus sorafenib.

## Discussion

In this study, we performed a cost-effectiveness analysis of nivolumab versus sorafenib for the therapy of aHCC, and the results of this study showed that compared with sorafenib, nivolumab was associated with incremental survival of 0.32 QALYs and incremental cost of $69,762 per patient. The calculated ICER was $220,864/QALY. One-way sensitivity analyses revealed that the cost of sorafenib and nivolumab was the most sensitive factor on the ICER, suggesting that the option between sorafenib and nivolumab could be made based on sorafenib and nivolumab costs. When the price of sorafenib exceeded $0.93/mg or nivolumab was less than $24.23/mg, nivolumab was cost-effective at a WTP threshold of $150,000/QALY. In this study, nivolumab was unlikely to be a cost-effective option at a WTP threshold of $150,000/QALY compared with sorafenib for the therapy of aHCC. According to the results of comprehensive deterministic and probabilistic sensitivity analyses, the results of this model are robust. The cost-effectiveness acceptability curves revealed that the probability of nivolumab being cost-effective was 10.38% at the WTP threshold of $150,000/QALY.

The cost-effectiveness of the therapy is substantially affected by the WTP threshold. A total of $100,000 or $150,000/QALY has been recommended as the WTP threshold in the United States (Bae and Mullins, 2014; Neumann et al., 2014). The ICERs of cancer drugs are often higher than those of other drugs. Even so, the Food and Drug Administration still approves new drugs to treat tumors based on their effectiveness in the United States. Many new drugs are used to treat tumors, despite an ICER greater than $100,000 or $150,000/QALY. An ICER of $220,864/QALY for nivolumab was shown in this study compared with sorafenib, suggesting that the ICER was higher than the WTP thresholds of $150,000/QALY. This result does not suggest antithesis to the use of nivolumab among patients with aHCC, but rather suggests that policymakers can maximize health gains by spending more resources on more cost-effective interventions (Neumann et al., 2014).

Because the cost of immune checkpoint inhibitor development is high, their prices are often high (Siddiqui and Rajkumar, 2012). Thus, it is common to see that an immune checkpoint inhibitor is not cost-effective as mentioned in the published literature (Verma et al., 2018). A study compared the cost-effectiveness of nivolumab with docetaxel in recurrent metastatic head and neck squamous cell carcinoma (HNSCC); although nivolumab exhibits clinical benefit in HNSCC treatment, it is not cost-effective based on the list price (Zargar et al., 2018).

To the best of our knowledge, this study is the first to conduct cost-effectiveness analyses of nivolumab versus sorafenib as first-line treatment for aHCC. Previously, immune checkpoint inhibitors have been discussed for the treatment of other malignant neoplasms, such as lung cancer, head and neck cancers, renal cell cancer, and melanoma (Verma et al., 2018). The clinical importance of this study is worth discussing. If the government successfully negotiates with pharmaceutical companies, the price of the drug may be reduced so that nivolumab can be cost-effective (Siddiqui and Rajkumar, 2012). As shown in this study, at a WTP threshold of $150,000/QALY, when the cost of nivolumab was less than $24.23/mg or the cost of sorafenib exceeded $0.93/mg, nivolumab was cost-effective.

The advantages of this study are worth noting. First, to our knowledge, this is the first assessment to evaluate the cost-effectiveness of nivolumab for the treatment for aHCC by combining the latest randomized clinical trial with a partitioned survival model. Second, compared to sorafenib treatment, the price is favorable, and cost-effectiveness was also estimated for nivolumab treatment among patients with aHCC. Third, patients and physicians may benefit from the economic information of subgroups when tailoring treatment decisions.

There were some limitations to this analysis. First, health outcomes that exceeded the follow-up time of the CheckMate 459 trial were assumed by fitting parametric distributions to the reported K-M OS and PFS data, which may have resulted in uncertainty in the model outputs. This limitation may not be a major factor according to the sensitivity analysis results, indicating that this finding is generally robust. Second, the CheckMate 459 trial is a phase 3 randomized clinical trial, and the parameters in the model are based on its results. Thus, the cost and effectiveness of the results may have been affected by biases within the trial. For example, the patients with aHCC enrolled in the CheckMate 459 trial were generally healthier than the general population of patients with aHCC. In addition, compared to patients in real-world practice, those who participate in clinical trials generally have better adherence to treatment.

## Conclusion

From the third-party payer perspective in the United States, this study suggests that at a WTP threshold of $150,000/QALY and under current drug pricing, nivolumab was unlikely to be considered cost-effective as first-line treatment for patients with aHCC compared with standard treatment with sorafenib. A substantial price reduction for nivolumab may result in favorable economic outcomes. Economic outcomes may be improved by tailoring individual treatments based on patient factors. These results may help clinicians to use appropriate treatments for patients with aHCC.

## Data Availability

The original contributions presented in the study are included in the article/Supplementary Material; further inquiries can be directed to the corresponding author.

## References

[B1] Amdahl J. Diaz J. Park J. Nakhaipour H. R. Delea T. E. (2016). Cost-effectiveness of Pazopanib Compared with Sunitinib in Metastatic Renal Cell Carcinoma in Canada. Curr. Oncol. 23 (4), e340–54. 10.3747/co.23.2244 27536183 PMC4974040

[B2] Bae Y. H. Mullins C. D. (2014). Do value Thresholds for Oncology Drugs Differ from Nononcology Drugs? J. Manag. Care Spec. Pharm. 20 (11), 1086–1092. 10.18553/jmcp.2014.20.11.1086 25351969 PMC10441029

[B3] Barzey V. Atkins M. B. Garrison L. P. Asukai Y. Kotapati S. Penrod J. R. (2013). Ipilimumab in 2nd Line Treatment of Patients with Advanced Melanoma: a Cost-Effectiveness Analysis. J. Med. Econ. 16 (2), 202–212. 10.3111/13696998.2012.739226 23057750

[B4] Borghaei H. Paz-Ares L. Horn L. Spigel D. R. Steins M. Ready N. E. (2015). Nivolumab versus Docetaxel in Advanced Nonsquamous Non-small-cell Lung Cancer. N. Engl. J. Med. 373 (17), 1627–1639. 10.1056/NEJMoa1507643 26412456 PMC5705936

[B5] Brahmer J. Reckamp K. L. Baas P. Crinò L. Eberhardt W. E. Poddubskaya E. (2015). Nivolumab versus Docetaxel in Advanced Squamous-Cell Non-small-cell Lung Cancer. N. Engl. J. Med. 373 (2), 123–135. 10.1056/NEJMoa1504627 26028407 PMC4681400

[B7] Centers for Medicare & Medicaid Services. (2022). ASP Drug Pricing Files. Available at: https://www.cms.gov/medicare/medicare-part-b-drug-average-sales-price/2022-asp-drug-pricing-files , (Accessed 20 March 2022).

[B8] Cheung K. S. Lam L. K. Seto W. K. Leung W. K. (2021). Use of Antibiotics during Immune Checkpoint Inhibitor Treatment Is Associated with Lower Survival in Hepatocellular Carcinoma. Liver Cancer 10 (6), 606–614. 10.1159/000518090 34950183 PMC8647068

[B9] El-Khoueiry A. B. Sangro B. Yau T. Crocenzi T. S. Kudo M. Hsu C. (2017). Nivolumab in Patients with Advanced Hepatocellular Carcinoma (CheckMate 040): an Open-Label, Non-comparative, Phase 1/2 Dose Escalation and Expansion Trial. Lancet 389 (10088), 2492–2502. 10.1016/s0140-6736(17)31046-2 28434648 PMC7539326

[B10] Forner A. Reig M. Bruix J. (2018). Hepatocellular Carcinoma. Lancet 391 (10127), 1301–1314. 10.1016/s0140-6736(18)30010-2 29307467

[B11] GetData Graph Digitizer. (2022). Digitizing Software. Digitize Scanned Graphs and Get Original (X,y) Data. Available at: http://getdata-graph-digitizer.com , (Accessed 20 March 2022).

[B12] Gordan J. D. Kennedy E. B. Abou-Alfa G. K. Beg M. S. Brower S. T. Gade T. P. (2020). Systemic Therapy for Advanced Hepatocellular Carcinoma: ASCO Guideline. J. Clin. Oncol. 38 (36), JCO2002672–4345. 10.1200/jco.20.02672 33197225

[B13] Guyot P. Ades A. E. Ouwens M. J. Welton N. J. (2012). Enhanced Secondary Analysis of Survival Data: Reconstructing the Data from Published Kaplan-Meier Survival Curves. BMC Med. Res. Methodol. 12, 9. 10.1186/1471-2288-12-9 22297116 PMC3313891

[B14] Hornberger J. Hirsch F. R. Li Q. Page R. D. (2015). Outcome and Economic Implications of Proteomic Test-Guided Second- or Third-Line Treatment for Advanced Non-small Cell Lung Cancer: Extended Analysis of the PROSE Trial. Lung Cancer 88 (2), 223–230. 10.1016/j.lungcan.2015.03.006 25804732

[B15] Husereau D. Drummond M. Augustovski F. de Bekker-Grob E. Briggs A. H. Carswell C. (2022). Consolidated Health Economic Evaluation Reporting Standards 2022 (CHEERS 2022) Statement: Updated Reporting Guidance for Health Economic Evaluations. Bmj 376, e067975. 10.1136/bmj-2021-067975 35017145 PMC8749494

[B16] Kacker S. Ness P. M. Savage W. J. Frick K. D. McCullough J. King K. E. (2013). The Cost-Effectiveness of Platelet Additive Solution to Prevent Allergic Transfusion Reactions. Transfusion 53 (11), 2609–2618. 10.1111/trf.12095 23363552 PMC3650119

[B17] Kudo M. Finn R. S. Qin S. Han K. H. Ikeda K. Piscaglia F. (2018). Lenvatinib versus Sorafenib in First-Line Treatment of Patients with Unresectable Hepatocellular Carcinoma: a Randomised Phase 3 Non-inferiority Trial. Lancet 391 (10126), 1163–1173. 10.1016/s0140-6736(18)30207-1 29433850

[B18] Neumann P. J. Cohen J. T. Weinstein M. C. (2014). Updating Cost-Effectiveness-Tthe Curious Resilience of the $50,000-Per-QALY Threshold. N. Engl. J. Med. 371 (9), 796–797. 10.1056/NEJMp1405158 25162885

[B19] Park J. W. Chen M. Colombo M. Roberts L. R. Schwartz M. Chen P. J. (2015). Global Patterns of Hepatocellular Carcinoma Management from Diagnosis to Death: the BRIDGE Study. Liver Int. 35 (9), 2155–2166. 10.1111/liv.12818 25752327 PMC4691343

[B20] Patel D. A. Holdford D. A. Edwards E. Carroll N. V. (2011). Estimating the Economic Burden of Food-Induced Allergic Reactions and Anaphylaxis in the United States. J. Allergy Clin. Immunol. 128 (1), 110. 10.1016/j.jaci.2011.03.013 21489610

[B21] Pei R. Shi Y. Lv S. Dai T. Zhang F. Liu S. (2021). Nivolumab vs Pembrolizumab for Treatment of US Patients with Platinum-Refractory Recurrent or Metastatic Head and Neck Squamous Cell Carcinoma: A Network Meta-Analysis and Cost-Effectiveness Analysis. JAMA Netw. Open 4 (5), e218065. 10.1001/jamanetworkopen.2021.8065 33956130 PMC8103222

[B22] RED BOOK online. (2022). IBM Micromedex; IBM Corporation. Available at: http://www.micromedexsolutions.com , (Accessed 20 March 2022).

[B23] Sanders G. D. Neumann P. J. Basu A. Brock D. W. Feeny D. Krahn M. (2016). Recommendations for Conduct, Methodological Practices, and Reporting of Cost-Effectiveness Analyses: Second Panel on Cost-Effectiveness in Health and Medicine. Jama 316 (10), 1093–1103. 10.1001/jama.2016.12195 27623463

[B24] Shlomai A. Leshno M. Goldstein D. A. (2018). Regorafenib Treatment for Patients with Hepatocellular Carcinoma Who Progressed on Sorafenib-A Cost-Effectiveness Analysis. PLoS One 13 (11), e0207132. 10.1371/journal.pone.0207132 30408106 PMC6224101

[B25] Siddiqui M. Rajkumar S. V. (2012). The High Cost of Cancer Drugs and what We Can Do about it. Mayo Clin. Proc. 87 (10), 935–943. 10.1016/j.mayocp.2012.07.007 23036669 PMC3538397

[B26] Soto-Perez-de-Celis E. Aguiar P. N. Cordón M. L. Chavarri-Guerra Y. Lopes G. L. (2019). Cost-Effectiveness of Cabozantinib in the Second-Line Treatment of Advanced Hepatocellular Carcinoma. J. Natl. Compr. Canc Netw. 17 (6), 669–675. 10.6004/jnccn.2018.7275 31200357

[B27] Su D. Wu B. Shi L. (2021). Cost-effectiveness of Atezolizumab Plus Bevacizumab vs Sorafenib as First-Line Treatment of Unresectable Hepatocellular Carcinoma. JAMA Netw. Open 4 (2), e210037. 10.1001/jamanetworkopen.2021.0037 33625508 PMC7905498

[B28] Tom’s Inflation Calculator. (2022). Medical-Care Inflation. Available at: https://www.halfhill.com/inflation_js.html , (Accessed 20 March 2022).

[B29] Vaidya A. Severens J. L. Bongaerts B. W. Cleutjens K. B. Nelemans P. J. Hofstra L. (2014). High-sensitive Troponin T Assay for the Diagnosis of Acute Myocardial Infarction: an Economic Evaluation. BMC Cardiovasc Disord. 14, 77. 10.1186/1471-2261-14-77 24927776 PMC4065542

[B30] Verma V. Sprave T. Haque W. Simone C. B. 2nd Chang J. Y. Welsh J. W. (2018). A Systematic Review of the Cost and Cost-Effectiveness Studies of Immune Checkpoint Inhibitors. J. Immunother. Cancer 6 (1), 128. 10.1186/s40425-018-0442-7 30470252 PMC6251215

[B31] Weber J. S. D'Angelo S. P. Minor D. Hodi F. S. Gutzmer R. Neyns B. (2015). Nivolumab versus Chemotherapy in Patients with Advanced Melanoma Who Progressed after Anti-CTLA-4 Treatment (CheckMate 037): a Randomised, Controlled, Open-Label, Phase 3 Trial. Lancet Oncol. 16 (4), 375–384. 10.1016/s1470-2045(15)70076-8 25795410

[B32] Williams C. Lewsey J. D. Mackay D. F. Briggs A. H. (2017). Estimation of Survival Probabilities for Use in Cost-Effectiveness Analyses: A Comparison of a Multi-State Modeling Survival Analysis Approach with Partitioned Survival and Markov Decision-Analytic Modeling. Med. Decis. Mak. 37 (4), 427–439. 10.1177/0272989x16670617 PMC5424853 27698003

[B33] Wilson L. Huang W. Chen L. Ting J. Cao V. (2017). Cost Effectiveness of Lenvatinib, Sorafenib and Placebo in Treatment of Radioiodine-Refractory Differentiated Thyroid Cancer. Thyroid 27 (8), 1043–1052. 10.1089/thy.2016.0572 28486081

[B34] Yau T. Park J. W. Finn R. S. Cheng A. L. Mathurin P. Edeline J. (2022). Nivolumab versus Sorafenib in Advanced Hepatocellular Carcinoma (CheckMate 459): a Randomised, Multicentre, Open-Label, Phase 3 Trial. Lancet Oncol. 23 (1), 77–90. 10.1016/s1470-2045(21)00604-5 34914889

[B35] Zakeri N. Hall A. Swadling L. Pallett L. J. Schmidt N. M. Diniz M. O. (2022). Characterisation and Induction of Tissue-Resident Gamma Delta T-Cells to Target Hepatocellular Carcinoma. Nat. Commun. 13 (1), 1372. 10.1038/s41467-022-29012-1 35296658 PMC8927126

[B36] Zargar M. McFarlane T. Chan K. K. W. Wong W. W. L. (2018). Cost-Effectiveness of Nivolumab in Recurrent Metastatic Head and Neck Squamous Cell Carcinoma. Oncologist 23 (2), 225–233. 10.1634/theoncologist.2017-0277 29021380 PMC5813741

