# Supplementary material for: Nivolumab Versus Sorafenib as First-Line Therapy for Advanced Hepatocellular Carcinoma: A Cost-Effectiveness Analysis
Source: Front Pharmacol. 2022 Jul 19;13:906956. doi: 10.3389/fphar.2022.906956 (PMC9343987; doi:10.3389/fphar.2022.906956)
Supplement: Supplementary file 1 [file DataSheet1.DOCX]

**Supplementary Content**

**Supplementary Figure 1.** Model Fitting Analysis

**Supplementary Figure 2.** Tornado Diagram of One-Way Sensitivity Analyses

**Supplementary Figure 3.** Impacts of Key Factors on ICER

**Supplementary Table 1.** CHEERS Checklist

**Supplementary Table 2.** Evaluated Parameters and Values of AIC and BIC

**Supplementary Table 3.** Probability and Costs Associated with Adverse Events (Grade ≥3)

**Supplementary Figure 1.** Model Fitting Analysis

To obtain the best model fit, the following investigations were carried out using nivolumab or sorafenib as the model fit baseline, respectively. Based on AIC and BIC (Supplementary Table 1), lognormal was used to fit the OS and PFS K-M of nivolumab and sorafenib, respectively.

(A) Model-fitted versus original K-M curves for nivolumab.


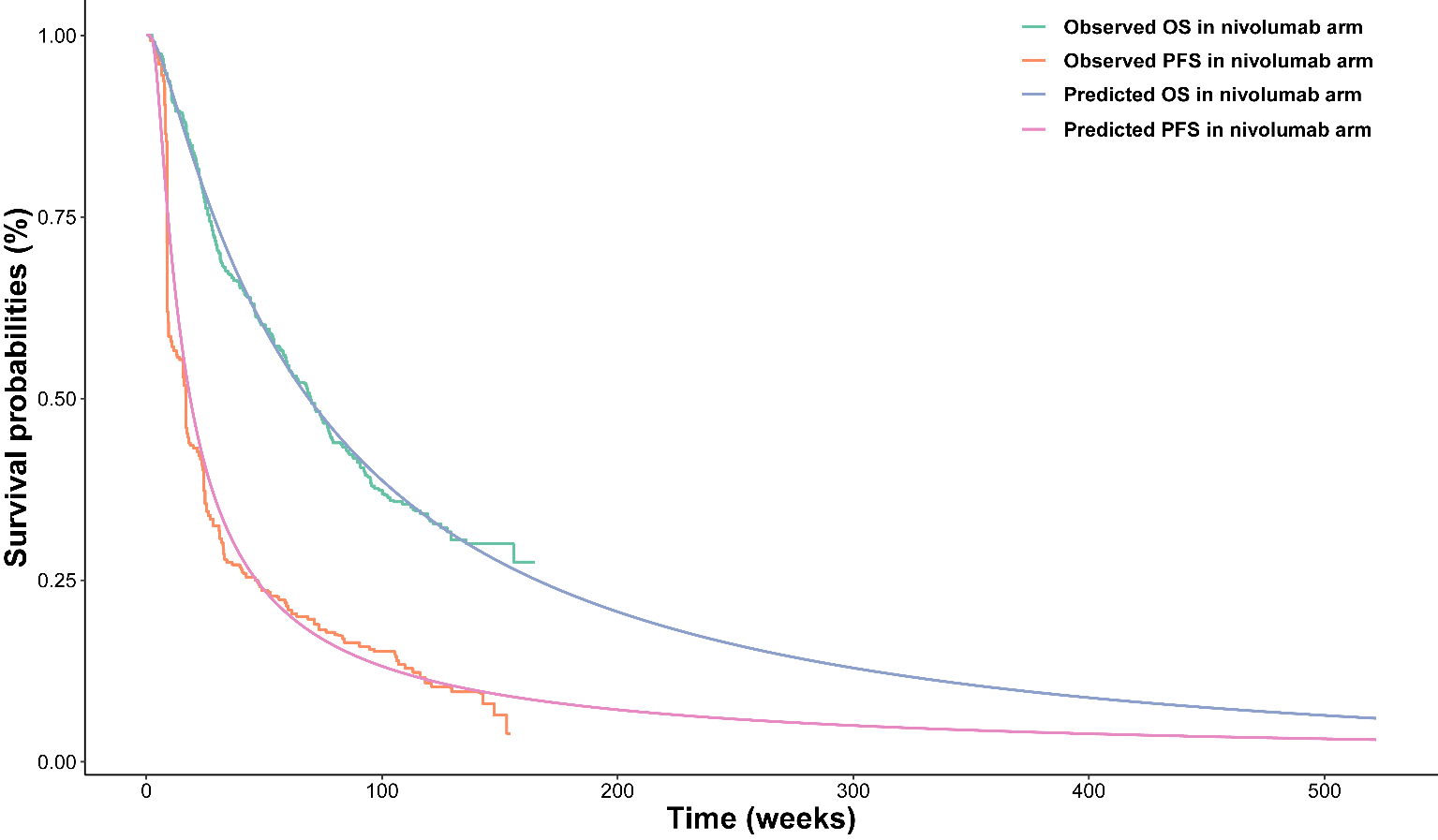


(B) Model-fitted versus original K-M curves for sorafenib.


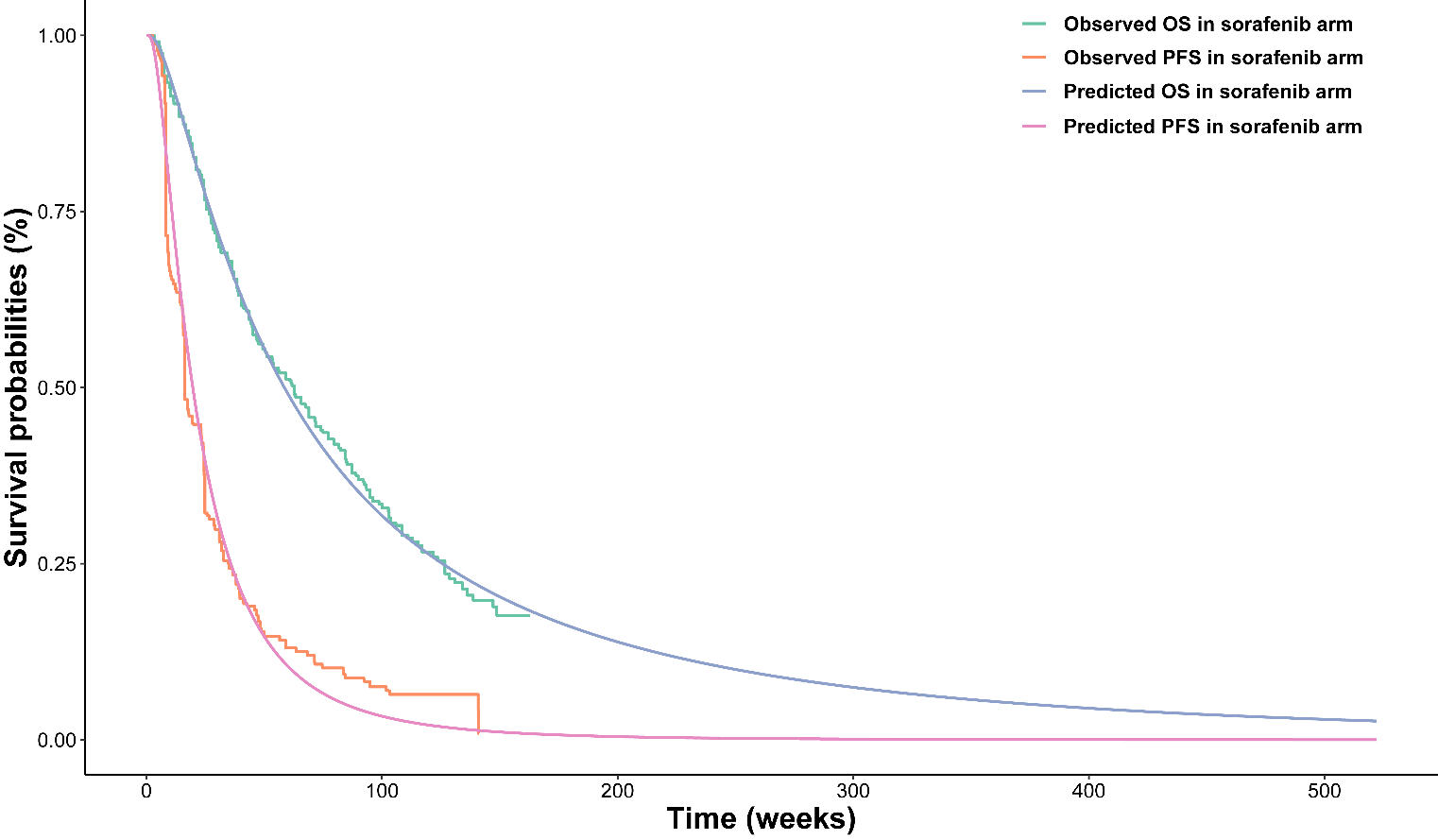


**Supplementary Figure 2.** Tornado Diagram of One-Way Sensitivity Analyses


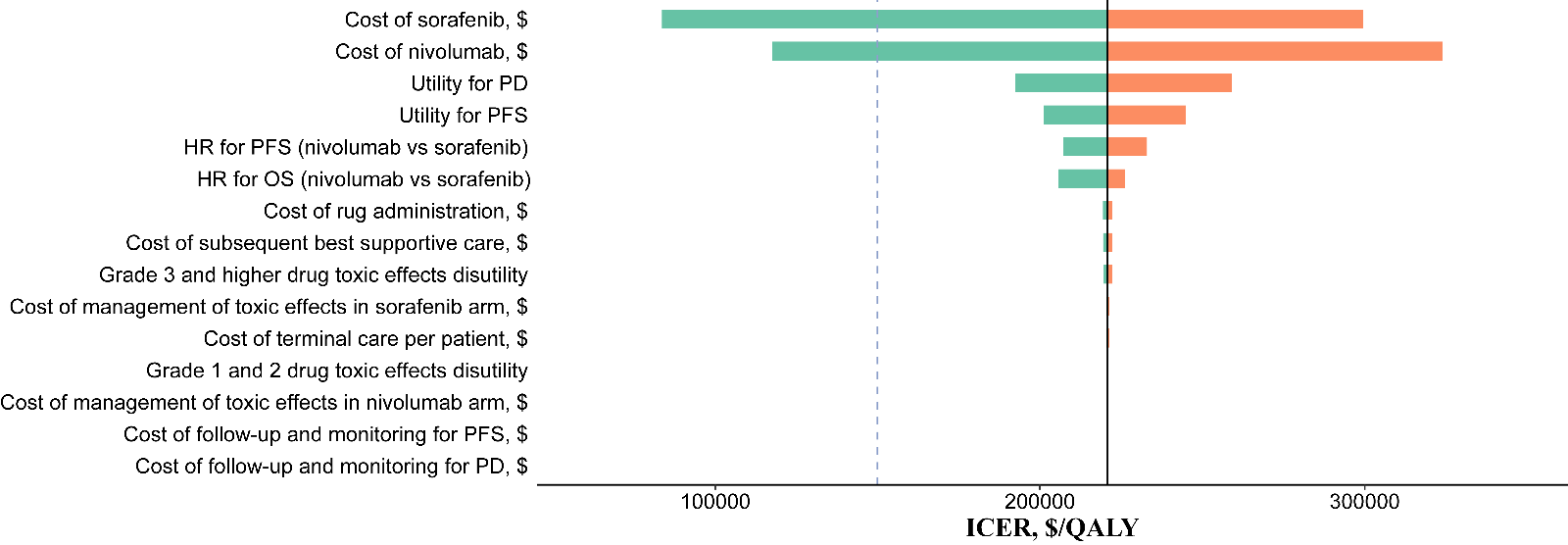


**Supplementary Figure 3.** Impacts of Key Factors on ICER

The diagrams show the impacts of key factors on the ICER (nivolumab versus sorafenib) for the treatment of aHCC. (A) represents the impacts of the cost of nivolumab; (B) represents the impacts of the cost of sorafenib. ICER: Incremental cost-effectiveness ratio; QALY: Quality-adjusted life year.

(A) represents the impacts of the cost of nivolumab


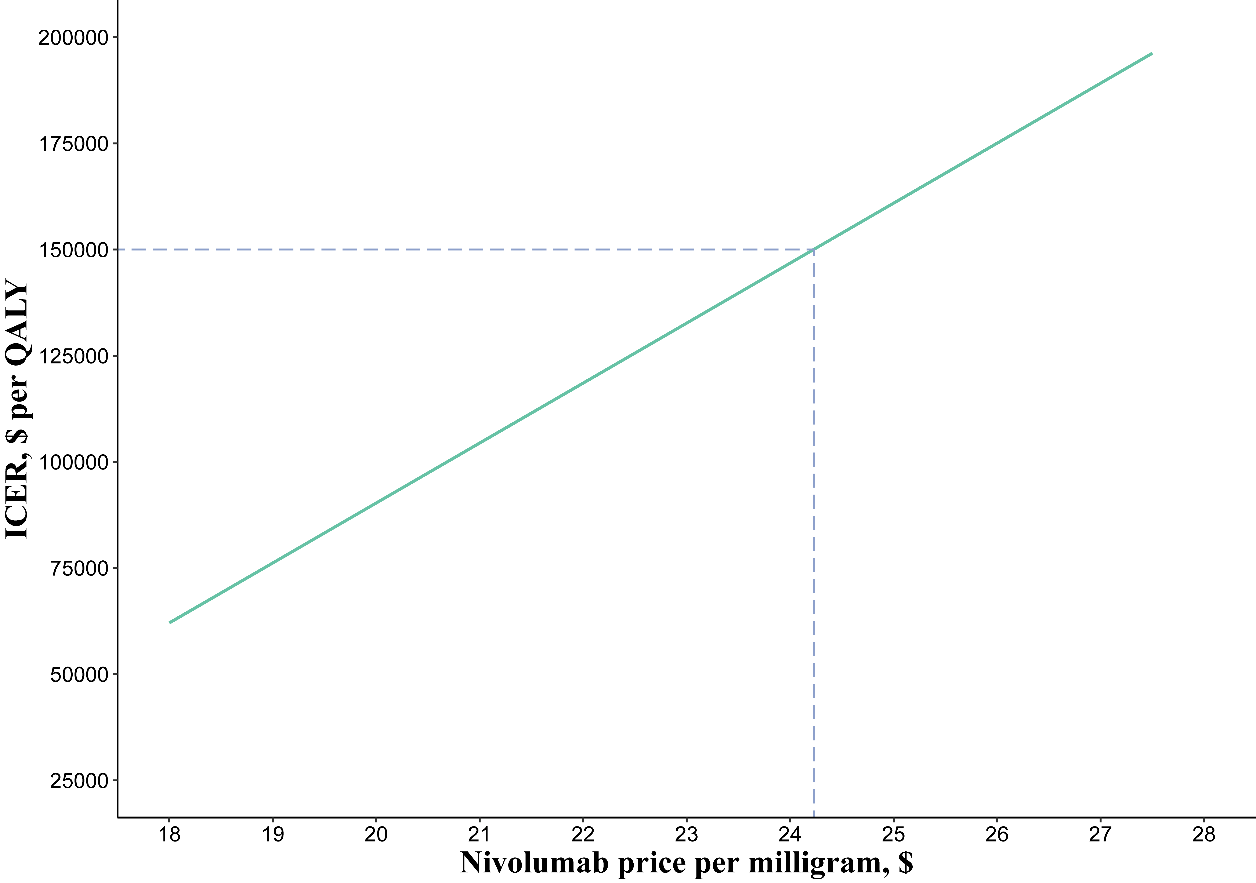


(B) represents the impacts of the cost of sorafenib


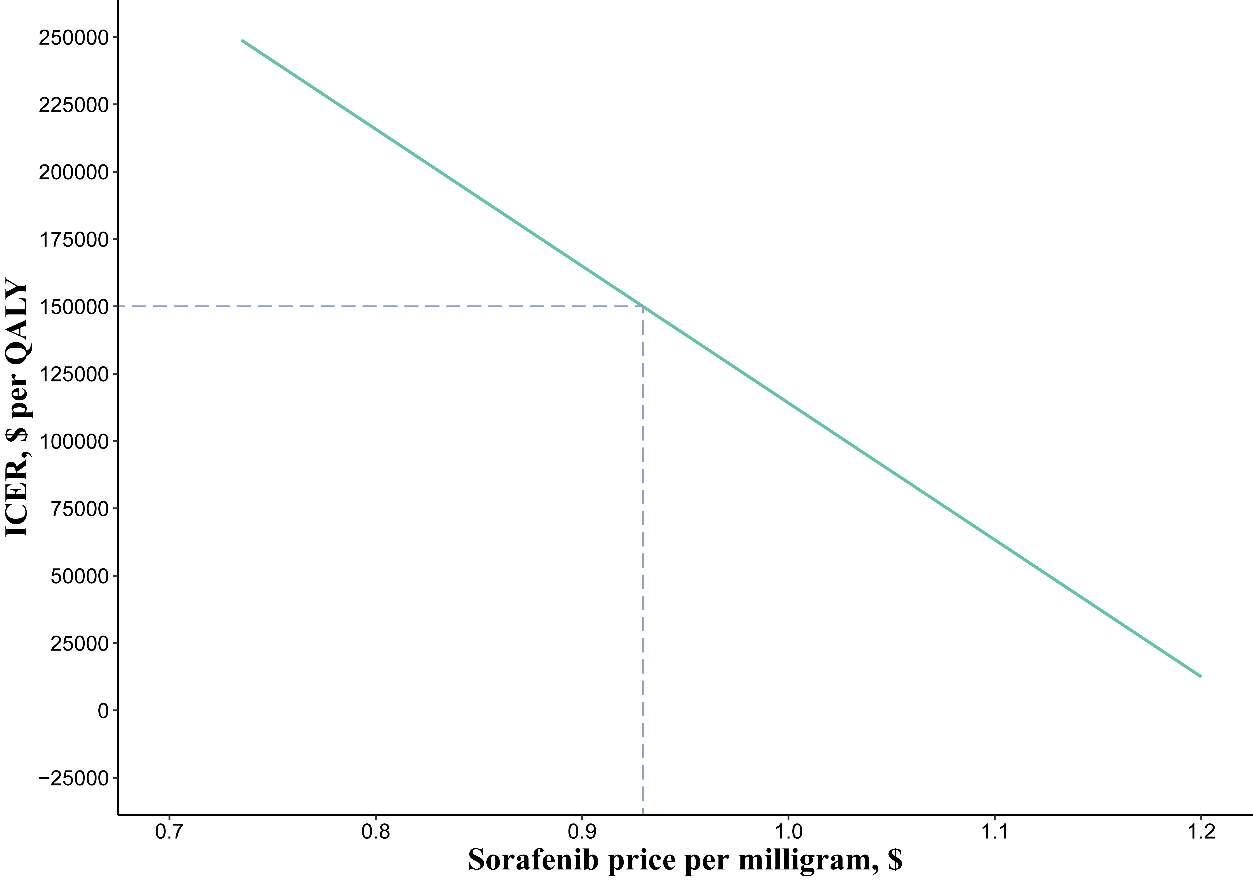


**Supplementary Table 1.** CHEERS Checklist

**CHEERS 2022 Checklist**

| **Topic** | **No.** | **Item** | **Location where item is reported** |
| --- | --- | --- | --- |
| **Title** |  |  |  |
|  | 1 | Identify the study as an economic evaluation and specify the interventions being compared. | Page 1 |
| **Abstract** |  |  |  |
|  | 2 | Provide a structured summary that highlights context, key methods, results, and alternative analyses. | Page 2-3 |
| **Introduction** |  |  |  |
| **Background and objectives** | 3 | Give the context for the study, the study question, and its practical relevance for decision making in policy or practice. | Page 4-5 |
| **Methods** |  |  |  |
| **Health economic analysis plan** | 4 | Indicate whether a health economic analysis plan was developed and where available. | NA |
| **Study population** | 5 | Describe characteristics of the study population (such as age range, demographics, socioeconomic, or clinical characteristics). | Page 5 |
| **Setting and location** | 6 | Provide relevant contextual information that may influence findings. | Page 5 |
| **Comparators** | 7 | Describe the interventions or strategies being compared and why chosen. | Page 5 |
| **Perspective** | 8 | State the perspective(s) adopted by the study and why chosen. | Page 5 |
| **Time horizon** | 9 | State the time horizon for the study and why appropriate. | Page 6 |
| **Discount rate** | 10 | Report the discount rate(s) and reason chosen. | Page 7 |
| **Selection of outcomes** | 11 | Describe what outcomes were used as the measure(s) of benefit(s) and harm(s). | Page 7-8 |
| **Measurement of outcomes** | 12 | Describe how outcomes used to capture benefit(s) and harm(s) were measured. | Page 7-8 |
| **Valuation of outcomes** | 13 | Describe the population and methods used to measure and value outcomes. | Page 7-8 |
| **Measurement and valuation of resources and costs** | 14 | Describe how costs were valued. | Page 6-7 |
| **Currency, price date, and conversion** | 15 | Report the dates of the estimated resource quantities and unit costs, plus the currency and year of conversion. | Page 6-7 |
| **Rationale and description of model** | 16 | If modelling is used, describe in detail and why used. Report if the model is publicly available and where it can be accessed. | Page 5-6 |
| **Analytics and assumptions** | 17 | Describe any methods for analysing or statistically transforming data, any extrapolation methods, and approaches for validating any model used. | Page 7-8 |
| **Characterising heterogeneity** | 18 | Describe any methods used for estimating how the results of the study vary for subgroups. | Page 8 |
| **Characterising distributional effects** | 19 | Describe how impacts are distributed across different individuals or adjustments made to reflect priority populations. | Page 8 |
| **Characterising uncertainty** | 20 | Describe methods to characterise any sources of uncertainty in the analysis. | Page 8 |
| **Approach to engagement with patients and others affected by the study** | 21 | Describe any approaches to engage patients or service recipients, the general public, communities, or stakeholders (such as clinicians or payers) in the design of the study. | Page 8 |
| **Results** |  |  |  |
| **Study parameters** | 22 | Report all analytic inputs (such as values, ranges, references) including uncertainty or distributional assumptions. | Page 8-9 |
| **Summary of main results** | 23 | Report the mean values for the main categories of costs and outcomes of interest and summarise them in the most appropriate overall measure. | Page 8-9 |
| **Effect of uncertainty** | 24 | Describe how uncertainty about analytic judgments, inputs, or projections affect findings. Report the effect of choice of discount rate and time horizon, if applicable. | Page 8-9 |
| **Effect of engagement with patients and others affected by the study** | 25 | Report on any difference patient/service recipient, general public, community, or stakeholder involvement made to the approach or findings of the study | Page 8-9 |
| **Discussion** |  |  |  |
| **Study findings, limitations, generalisability, and current knowledge** | 26 | Report key findings, limitations, ethical or equity considerations not captured, and how these could affect patients, policy, or practice. | Page 10-12 |
| **Other relevant information** |  |  |  |
| **Source of funding** | 27 | Describe how the study was funded and any role of the funder in the identification, design, conduct, and reporting of the analysis | Page 12 End of manuscript |
| **Conflicts of interest** | 28 | Report authors conflicts of interest according to journal or International Committee of Medical Journal Editors requirements. | Page 13 End of manuscript |

**Supplementary Table 2.** Evaluated Parameters and Values of AIC and BIC

| **Strategies** | **Distributions** | **Parameters** | **est** | **se** | **L95%** | **U95%** | **AIC** | **BIC** |
| --- | --- | --- | --- | --- | --- | --- | --- | --- |
| **Results of OS** | | | | | | | | |
| Nivolumab | Exponential | rate | 0.00917513 | 0.000586 | 0.008095 | 0.010399 | 2790.717 | 2794.633 |
|  | WeibullPH | shape | 1.00104336 | 0.054939 | 0.898954 | 1.114726 | 2792.716 | 2800.549 |
|  |  | scale | 0.00913175 | 0.002352 | 0.005512 | 0.015129 |  |  |
|  | Gamma | shape | 1.05573823 | 0.082161 | 0.906385 | 1.229702 | 2792.237 | 2800.07 |
|  |  | rate | 0.00984228 | 0.001159 | 0.007814 | 0.012398 |  |  |
|  | Lognormal | meanlog | 4.23336 | 0.073735 | 4.08885 | 4.37788 | **2769.066** | **2776.898** |
|  |  | sdlog | 1.30053 | 0.062777 | 1.18313 | 1.42958 |  |  |
|  | Gompertz | shape | -0.0038553 | 0.001716 | -0.00722 | -0.00049 | 2787.485 | 2795.317 |
|  |  | rate | 0.01114533 | 0.001158 | 0.009092 | 0.013662 |  |  |
|  | Log-logistic | shape | 1.3062 | 0.069696 | 1.1765 | 1.4502 | 2775.415 | 2783.247 |
|  |  | scale | 68.6565 | 4.952107 | 59.6054 | 79.082 |  |  |
|  | Generalized gamma | mu | 4.097688 | 0.153747 | 3.796349 | 4.399027 | 2769.988 | 2781.737 |
|  |  | sigma | 1.347869 | 0.074487 | 1.209506 | 1.50206 |  |  |
|  |  | Q | -0.258387 | 0.248611 | -0.74566 | 0.228882 |  |  |
| Sorafenib | Exponential | rate | 0.0110526 | 0.000662 | 0.009829 | 0.012429 | 3073.842 | 3077.761 |
|  | WeibullPH | shape | 1.12799023 | 0.057042 | 1.021552 | 1.245519 | 3070.447 | 3078.285 |
|  |  | scale | 0.00621515 | 0.001645 | 0.0037 | 0.01044 |  |  |
|  | Gamma | shape | 1.2456152 | 0.093316 | 1.075512 | 1.442622 | 3067.684 | 3075.521 |
|  |  | rate | 0.0143464 | 0.001471 | 0.011735 | 0.017539 |  |  |
|  | Lognormal | meanlog | 4.07046 | 0.061772 | 3.94939 | 4.19153 | **3054.74** | **3062.578** |
|  |  | sdlog | 1.13148 | 0.05026 | 1.03714 | 1.2344 |  |  |
|  | Gompertz | shape | 0.00105256 | 0.001582 | -0.00205 | 0.004154 | 3075.404 | 3083.242 |
|  |  | rate | 0.010476 | 0.001062 | 0.008589 | 0.012778 |  |  |
|  | Log-logistic | shape | 1.5015 | 0.074526 | 1.36232 | 1.65491 | 3061.239 | 3069.077 |
|  |  | scale | 59.4209 | 3.652966 | 52.67577 | 67.02981 |  |  |
|  | Generalized gamma | mu | 4.1009414 | 0.123781 | 3.858335 | 4.34355 | 3056.661 | 3068.418 |
|  |  | sigma | 1.1204203 | 0.064436 | 1.000985 | 1.25411 |  |  |
|  |  | Q | 0.0642679 | 0.22796 | -0.38253 | 0.51106 |  |  |
| **Results of PFS** | | | | | | | | |
| Nivolumab | Exponential | rate | 0.0265074 | 0.001538 | 0.023658 | 0.0297 | 2752.417 | 2756.333 |
|  | WeibullPH | shape | 0.9028825 | 0.039173 | 0.829279 | 0.983019 | 2748.552 | 2756.385 |
|  |  | scale | 0.0386529 | 0.006229 | 0.028184 | 0.05301 |  |  |
|  | Gamma | shape | 0.9725276 | 0.068127 | 0.847761 | 1.115656 | 2754.257 | 2762.09 |
|  |  | rate | 0.0256777 | 0.002552 | 0.021132 | 0.031201 |  |  |
|  | Lognormal | meanlog | 3.05233 | 0.059616 | 2.93549 | 3.16918 | **2596.018** | **2607.767** |
|  |  | sdlog | 1.10025 | 0.046349 | 1.01306 | 1.19495 |  |  |
|  | Gompertz | shape | -0.0138501 | 0.002258 | -0.01828 | -0.00942 | 2707.972 | 2715.805 |
|  |  | rate | 0.0396322 | 0.003111 | 0.033981 | 0.046223 |  |  |
|  | Log-logistic | shape | 1.55529 | 0.07436 | 1.41617 | 1.70808 | 2654.702 | 2662.535 |
|  |  | scale | 19.01204 | 1.149797 | 16.88691 | 21.4046 |  |  |
|  | Generalized gamma | mu | 2.513049 | 0.075902 | 2.364284 | 2.661815 | 2649.239 | 2657.071 |
|  |  | sigma | 0.929204 | 0.042677 | 0.849212 | 1.01673 |  |  |
|  |  | Q | -1.104217 | 0.129687 | -1.3584 | -0.85004 |  |  |
| Sorafenib | Exponential | rate | 0.0327448 | 0.00195 | 0.029138 | 0.036799 | 2494.321 | 2498.24 |
|  | WeibullPH | shape | 1.117051 | 0.047281 | 1.028121 | 1.213672 | 2489.93 | 2497.768 |
|  |  | scale | 0.021491 | 0.003918 | 0.015034 | 0.03072 |  |  |
|  | Gamma | shape | 1.4125485 | 0.10285 | 1.22469 | 1.629223 | 2475.677 | 2483.515 |
|  |  | rate | 0.0483875 | 0.004591 | 0.040176 | 0.058278 |  |  |
|  | Lognormal | meanlog | 2.981033 | 0.049272 | 2.884462 | 3.077604 | **2370.101** | **2381.858** |
|  |  | sdlog | 0.886551 | 0.037683 | 0.815687 | 0.963572 |  |  |
|  | Gompertz | shape | -0.00536637 | 0.002467 | -0.0102 | -0.00053 | 2491.169 | 2499.007 |
|  |  | rate | 0.03707741 | 0.002954 | 0.031718 | 0.043343 |  |  |
|  | Log-logistic | shape | 1.94105 | 0.094067 | 1.76517 | 2.13446 | 2405.316 | 2413.154 |
|  |  | scale | 18.57094 | 0.923621 | 16.84611 | 20.47237 |  |  |
|  | Generalized gamma | mu | 2.606382 | 0.074592 | 2.46018 | 2.752579 | 2399.358 | 2407.196 |
|  |  | sigma | 0.79817 | 0.038651 | 0.7259 | 0.877636 |  |  |
|  |  | Q | -0.911351 | 0.155201 | -1.21554 | -0.60716 |  |  |

**Abbreviation:** AIC, Akaike information criterion; BIC, Bayesian information criterion

**Supplementary Table 3.** Probability and Costs Associated with Adverse Events (Grade ≥3)

| Variable^a^ | Costs per event | | |  | Probabilities in nivolumab arm^b^ | | | Probabilities in sorafenib arm^b^ | | |
| --- | --- | --- | --- | --- | --- | --- | --- | --- | --- | --- |
|  | Baseline value | Range | Distribution | Reference | Baseline value | Range | Distribution | Baseline value | Range | Distribution |
| Hypertension | 15811 | 11858.26 to 19763.76 | Gamma | (Wilson et al., 2017) | 0 | NA | NA | 0.072 | 0.054 to 0.09 | Beta |
| Fatigue | 2895.23 | 1852.72 to 4165.82 | Gamma | (Barzey et al., 2013) | 0.008 | 0.006 to 0.01 | Beta | 0.019 | 0.014 to 0.024 | Beta |
| Diarrhea | 1084.48 | 694.08 to 1535.08 | Gamma | (Barzey et al., 2013) | 0.008 | 0.006 to 0.01 | Beta | 0.050 | 0.037 to 0.062 | Beta |
| Decreased appetite | 13843.92 | 10372.35 to 17304.88 | Gamma | (Wilson et al., 2017) | 0 | NA | NA | 0.017 | 0.012 to 0.021 | Beta |
| Rash | 305.99 | 229.48 to 382.48 | Gamma | (Hornberger et al., 2015) | 0 | NA | NA | 0.017 | 0.012 to 0.021 | Beta |
| Infusion-related reaction | 7454.24 | 5590.67 to 9317.79 | Gamma | (Patel et al., 2011; Kacker et al., 2013) | 0.060 | 0.045 to 0.075 | Beta | 0.036 | 0.027 to 0.045 | Beta |
| Palmar–plantar erythrodysesthesia syndrome | 9013.11 | 6759.83 to 11266.39 | Gamma | (Wilson et al., 2017) | 0.003 | 0.002 to 0.003 | Beta | 0.143 | 0.107 to 0.179 | Beta |
| Weight decreased | 757.44 | 568.08 to 946.8 | Gamma | (Wilson et al., 2017) | 0 | NA | NA | 0.011 | 0.008 to 0.014 | Beta |
| Nausea and vomiting | 37.06 | 27.8 to 46.33 | Gamma | (Wilson et al., 2017) | 0 | NA | NA | 0.011 | 0.008 to 0.014 | Beta |

^a^Our analysis only included and evaluated grade more than 3 treatment-related adverse events.

^b^Number within treatment arm: nivolumab (N=367), sorafenib (N=363).

**References**

Barzey, V., Atkins, M.B., Garrison, L.P., Asukai, Y., Kotapati, S., and Penrod, J.R. (2013). Ipilimumab in 2nd line treatment of patients with advanced melanoma: a cost-effectiveness analysis. *J Med Econ* 16(2), 202-212. doi: 10.3111/13696998.2012.739226.

Hornberger, J., Hirsch, F.R., Li, Q., and Page, R.D. (2015). Outcome and economic implications of proteomic test-guided second- or third-line treatment for advanced non-small cell lung cancer: extended analysis of the PROSE trial. *Lung Cancer* 88(2), 223-230. doi: 10.1016/j.lungcan.2015.03.006.

Kacker, S., Ness, P.M., Savage, W.J., Frick, K.D., McCullough, J., King, K.E., et al. (2013). The cost-effectiveness of platelet additive solution to prevent allergic transfusion reactions. *Transfusion* 53(11), 2609-2618. doi: 10.1111/trf.12095.

Patel, D.A., Holdford, D.A., Edwards, E., and Carroll, N.V. (2011). Estimating the economic burden of food-induced allergic reactions and anaphylaxis in the United States. *J Allergy Clin Immunol* 128(1), 110-115.e115. doi: 10.1016/j.jaci.2011.03.013.

Wilson, L., Huang, W., Chen, L., Ting, J., and Cao, V. (2017). Cost Effectiveness of Lenvatinib, Sorafenib and Placebo in Treatment of Radioiodine-Refractory Differentiated Thyroid Cancer. *Thyroid* 27(8), 1043-1052. doi: 10.1089/thy.2016.0572.
